# Hypoglycemia after Mitral Valve Repair in Dogs

**DOI:** 10.3390/vetsci11020079

**Published:** 2024-02-08

**Authors:** Yasuyuki Nii, Emi Takahashi, Miho Tabata, Shimon Furusato, Masaya Katsumata, Masami Uechi

**Affiliations:** 1 JASMINE Veterinary Cardiovascular Medical Center, Yokohama 224-0001, Japan; nii.yasuyuki@jasmine-vet.co.jp (Y.N.); takahashi.emi@jasmine-vet.co.jp (E.T.); furusato.shimon@jasmine-vet.co.jp (S.F.); 2 Laboratory of Nutrition of Veterinary Medicine, School of Veterinary Medicine, Azabu University, Sagamihara 252-5201, Japan; m-katsumata@azabu-u.ac.jp; 3 Arsci Inc., Yokohama 224-0001, Japan; tabata.miho@arsci.co.jp

**Keywords:** postoperative hypoglycemia, insulin, glucagon, glucose, CPB, MMVD, canine

## Abstract

**Simple Summary:**

Hypoglycemia has not been previously reported as a postoperative complication of mitral valve repair in dogs. However, the authors encountered cases of hypoglycemia after mitral valve repair in dogs. Therefore, this study aimed to determine the incidence of hypoglycemia in dogs after mitral valve repair and investigate its causes. This study revealed that the incidence of hypoglycemia after mitral valve repair was 14.2%, and plasma glucagon concentrations increased in these dogs, whereas serum insulin concentrations decreased compared with preoperative levels. Therefore, hyperinsulinemia or hypoglucagonemia does not appear to be the cause of postoperative hypoglycemia. Furthermore, the results of this study indicate that the risk factors for hypoglycemia were low body weight and asymptomatic myxomatous mitral valve disease. Monitoring blood glucose levels after mitral valve repair should be included in the standard hospitalization plan to prevent hypoglycemic emergencies in dogs.

**Abstract:**

Hypoglycemia has not been previously reported as a postoperative complication of mitral valve repair (MVR) in dogs; however, the authors have encountered cases of hypoglycemia after MVR. This study aimed to determine the incidence of hypoglycemia in dogs after MVR and investigate its causes. Blood glucose levels were measured at multiple timepoints in dogs undergoing MVR. Simultaneously, insulin and glucagon blood concentrations in dogs with hypoglycemia preoperatively and postoperatively were compared to verify the physiological responses to hypoglycemia. Furthermore, risk factors for hypoglycemia, using variables selected based on the characteristics of MVR and dogs undergoing MVR, were examined prospectively. The incidence of hypoglycemia after MVR was 14.2%, and plasma glucagon concentrations increased in these dogs (mean: 260 pg/mL and 644 pg/mL pre- and postoperatively, *p* < 0.001), whereas serum insulin concentrations decreased (median: 0.50 ng/mL and 0.29 ng/mL pre- and postoperatively, *p* = 0.002). Therefore, hyperinsulinemia or hypoglucagonemia is unlikely to be the cause of postoperative hypoglycemia. The identified risk factors for hypoglycemia included low body weight and asymptomatic myxomatous mitral valve disease. Monitoring blood glucose levels after MVR should be included in the standard hospitalization plan to prevent hypoglycemic emergencies in dogs.

## 1. Introduction

Blood glucose levels are regulated within an appropriate range, and disturbances in this mechanism lead to hyperglycemia or hypoglycemia. Postoperative hyperglycemia, a common problem in human medicine, results from hypermetabolic stress responses [1,2]. Insulin is often administered for perioperative glucose management to prevent such hyperglycemia and infection of surgical sites [3,4]. Moreover, in human cardiac surgery, excessive insulin administration is the primary cause of postoperative hypoglycemia. Postoperative hypoglycemia in patients without insulin administration is rare and has no known cause [5,6]. In veterinary medicine, postoperative hypoglycemia has been reported in insulinoma cases, where hypoglycemia was present before surgery [7,8], or in portosystemic shunts cases [9]. Previous studies have not reported hypoglycemia as a postoperative complication of mitral valve repair (MVR) in dogs [10]. However, the authors have previously encountered several cases of postoperative hypoglycemia in dogs undergoing MVR.

This study aimed to investigate the incidence and causes of postoperative hypoglycemia in dogs that underwent MVR. Blood insulin and glucagon concentrations, which play a major role in regulating blood glucose levels, were compared pre- and postoperatively to examine the physiological responses to hypoglycemia. Furthermore, a multivariate logistic regression analysis was performed using variables related to the characteristics of MVR and to those of the dogs that underwent MVR to verify the risk factors for hypoglycemia.

## 2. Materials and Methods

### 2.1. Dogs

The study procedures were reviewed and approved by the Ethics Committee of the Japan Animal Specialty Medical Institute, Inc. (Yokohama, Japan, approval number: 181025-3). All procedures were performed in accordance with the institutional guidelines for animal welfare.

The study group comprised client-owned dogs that underwent MVR between January and May 2019. The owner’s consent for the academic utilization of data was obtained for all cases. All dogs were diagnosed with myxomatous mitral valve disease (MMVD), and its severity was classified according to the guidelines of the American College of Veterinary Internal Medicine (ACVIM) [11]. The criterion for surgical eligibility was MMVD severity of stage B2 or higher. Additionally, these dogs underwent physical examinations, noninvasive blood pressure measurements, electrocardiography, chest and abdominal radiography, echocardiography, abdominal ultrasound examination, urinalysis, and blood tests to ensure the absence of severe ailments other than cardiac diseases. Diabetes mellitus was excluded by conducting blood tests and urinalysis; malignancy was excluded by physical examination and imaging diagnosis; and liver failure, Cushing’s syndrome, and Addison’s syndrome were excluded by blood tests and imaging diagnosis. Adrenocorticotropic hormone (ACTH) stimulation tests were conducted for suspected cases of Cushing’s syndrome and Addison’s syndrome. Furthermore, these dogs were not administered steroids that could potentially affect glucose metabolism. All dogs underwent a fasting period of approximately 12 h preoperatively.

The authors gathered individual data for each case, including data on breed, age, body weight, sex, MMVD severity (categorized into two groups: asymptomatic without congestive heart failure development and symptomatic with congestive heart failure development), body condition score (BCS), muscle condition score (MCS), hemodilution rate, presence of allogeneic blood transfusion, presence of blood collection for autologous blood transfusion, presence of autologous washed red blood cell (RBC) transfusion, in/out fluid balance during cardiopulmonary bypass (CPB), CPB time, cardiac arrest time, time under anesthesia, and presence of postoperative opioid utilization.

BCS was evaluated on a 9-point scale, with a score of 4 or 5 considered ideal, ≤3 indicating thinness, and ≥6 indicating obesity. MCS was evaluated on a 4-point scale, with a score of 4 considered optimal and a score of ≤3 indicating decreased muscle mass.

### 2.2. Mitral Valve Repair [10,12,13]

MVR was performed using CPB under extracorporeal circulation and involved mitral annuloplasty and artificial chordal replacement. On the day of MVR, the administered cardiovascular medications comprised either pimobendan alone or a combination of pimobendan and loop diuretics. The anesthesia and surgical methods for MVR were as follows: glycopyrrolate (10 μg/kg, SC) was administered, and midazolam (0.3 mg/kg, IV) and fentanyl (5 μg/kg, IV) were administered as premedication. Subsequently, ketamine (5 mg/kg, IV) was administered to induce anesthesia, and endotracheal intubation was performed, followed by the attachment of vital monitors. Additionally, anesthesia was maintained using isoflurane (0.5–3%) intraoperatively, and perioperative analgesia was established by continuous rate infusion (CRI) of fentanyl (10–20 μg/kg/min). For invasive arterial and venous pressure measurements, a catheter was inserted into the dorsal pedal or tail artery and the saphenous or dorsal pedal vein. In cases where blood pressure was stable, blood collection for autologous blood transfusion was performed using anticoagulant citrate dextrose solution A (ACD-A) followed by the administration of heparin (200 IU/kg, IV). In cases with reduced blood pressure, additional crystalloid fluid volume or CRI of dobutamine (1–5 μg/kg/min) was administered. Moreover, after confirming effective anticoagulation, a cannula for blood perfusion was inserted into the carotid artery, and a cannula for blood drainage was placed into the right atrium through the jugular vein; CPB was initiated.

Thoracotomy was performed on the left side of the chest in the fourth or fifth intercostal space, and the pericardium was incised. A cannula for the infusion of the myocardial protection solution was positioned at the base of the aorta. The ascending aorta was clamped, and a myocardial protection solution was infused via the aortic cannula to induce cardiac arrest. The blood-based St. Thomas cardioplegia solution was used for myocardial protection. Infusion was maintained at intervals of every 5–10 min, even after cardiac arrest. Central cooling was employed to lower the body temperature to 28–30 °C. The left atrial appendage was incised, and artificial chordal replacement and mitral annuloplasty were performed. Furthermore, artificial chordal replacement was performed using polytetrafluoroethylene sutures as artificial chordae. Mitral annuloplasty was performed, involving the use of polytetrafluoroethylene sutures and pledgets in the region from the cranial to the caudal commissure.

The aortic clamp was released (declamped) after the closure of the left atrial appendage, and normal heart rhythm was resumed. After confirming that mitral regurgitation improved compared to the preoperative state through transesophageal echocardiography, the chest was closed following standard procedures.

After ensuring stable hemodynamics and rewarming, extracorporeal circulation was discontinued. Heparin reversal was achieved using a CRI of protamine (3 mg/kg). In cases of blood collection for autologous blood transfusion, venous reinfusion was performed after the discontinuation of extracorporeal circulation. The cannulae used for blood perfusion and drainage were removed. After all procedures were completed, the isoflurane concentration was gradually reduced to awaken the dog from anesthesia.

CPB time was defined as the duration from the initiation to the cessation of CPB. Cardiac arrest time was defined as the duration from aortic clamping to declamping. Time under anesthesia was defined as the duration from endotracheal intubation to extubation.

After extubation, postoperative care was provided while monitoring the vital signs in an oxygen-enriched environment. Chest drainage tubes were typically removed the following morning, when fluid drainage was reduced to a minimal amount. Additionally, the postoperative medications administered included cefazolin (20 mg/kg, IV) or cefalexin (20 mg/kg, PO), clopidogrel (2–4 mg/kg, PO), and dalteparin (100–150 IU/kg, SC). Postoperative pain management included opioids, based on the dogs’ conditions: CRI of fentanyl (3–6 μg/kg/h), single-dose butorphanol injection (0.2–0.4 mg/kg, IV), and single-dose buprenorphine injection (0.01–0.02 mg/kg, IV). The dogs were discharged after the presence of postoperative complications was ruled out by conducting physical examinations, noninvasive blood pressure measurements, electrocardiography, chest radiography, echocardiography, and blood tests.

### 2.3. Cardiopulmonary Bypass [12,13]

The CPB was assembled using an artificial lung, reservoir, roller pump, and a circuit connected to a dog, with the circuit primed with a filling solution. The filling solution was composed of 20% D-mannitol (5 mL/kg), 7% sodium bicarbonate (2 mL/kg), sodium heparin (500 IU/kg), and Ringer’s acetate solution (120–170 mL), excluding glucose. The blood dilution caused by the filling solution at the initiation of CPB was calculated as the hemodilution rate (Equations (1) and (2)). During CPB, crystalloid fluid (without glucose, similar to the filling solution) was added as needed while observing the blood pressure and fluid level in the reservoir. Moreover, the amount of crystalloid fluid and cardioprotective solution (St. Thomas Hospital solution) added during CPB was recorded as the infusion volume. In cases where anemia progressed owing to blood dilution, allogeneic blood, preserved with the mannitol adenine phosphate (MAP) solution, was administered.

To evaluate fluid volume at the end of CPB, the in/out fluid balance during CPB was calculated (Equation (3)). The fluid remaining in the reservoir after CPB was centrifuged, cleaned, and stored as centrifugal reservoir blood and used for autologous washed RBC transfusions if postoperative anemia developed.

(1) $$\mathrm{Hemodilution}\;\mathrm{rate}\;\left(\%\right)=\frac{\mathrm{Filling}\;\mathrm{solution}\;\mathrm{volume}\;\left(\mathrm{mL}\right)}{\;\mathrm{Filling}\;\mathrm{solution}\;\mathrm{volume}\;\left(\mathrm{mL}\right)+\mathrm{Circulating}\;\mathrm{blood}\;\mathrm{volume}\;\left(\mathrm{mL}\right)}\times 100$$

(2) $$\mathrm{Circulating}\;\mathrm{blood}\;\mathrm{volume}\;\left(\mathrm{mL}\right)=\mathrm{Body}\;\mathrm{weight}\left(\mathrm{kg}\right)\times 90$$

(3) $$\mathrm{In}/\mathrm{out}\mathrm{balance}\;\mathrm{during}\;\mathrm{CPB}\;\left(\mathrm{mL}/\mathrm{kg}\right)=\frac{\mathrm{Infusion}\;\mathrm{volume}\;\mathrm{during}\;\mathrm{CPB}\;\left(\mathrm{mL}\right)-\mathrm{Urine}\;\mathrm{volume}\;\mathrm{during}\;\mathrm{CPB}\;\left(\mathrm{mL}\right)}{\mathrm{Body}\;\mathrm{weight}\;\left(\mathrm{kg}\right)}$$

### 2.4. Blood Sampling

Blood samples were collected at four timepoints: preoperatively, after the completion of CPB, 6–14 h postoperatively, and 16–20 h postoperatively. If hypoglycemia was observed, the study was terminated, and a diet was administered or a therapeutic intervention was performed. Twenty hours postoperatively, all dogs were started on a diet, and blood samples were not collected thereafter.

### 2.5. Blood Biochemical Data

Blood glucose levels were measured in the hospital using the FUJI DRI-CHEM system, with a reference range of 75–128 mg/dL (4.2–7.1 mmol/L), and values below 75 mg/dL (4.2 mmol/L) were considered indicative of hypoglycemia [14]. The serum insulin and plasma glucagon concentrations were measured twice. The first measurement was performed preoperatively, and the second either when hypoglycemia was detected postoperatively or when hypoglycemia was not observed 16–20 h postoperatively. Serum insulin and plasma glucagon concentrations were measured by Fujifilm Monolith Co., Ltd. Mitaka, Japan (commercial serum insulin concentration measurement: chemiluminescent immunoassay method; plasma glucagon concentration measurement: radioimmunoassay method). The insulin/glucagon (I/G) ratio was calculated using serum insulin and plasma glucagon concentrations.

### 2.6. Statistical Analysis

Based on the presence of hypoglycemia, the obtained data were divided into hypoglycemia and comparison groups. Shapiro–Wilk tests were conducted, and normally distributed data are presented as mean (with standard deviation [SD]), whereas non-normally distributed data are presented as median (interquartile range [IQR]). Paired *t*-tests or Wilcoxon signed-rank tests were used to analyze the changes in insulin and glucagon concentrations and the I/G ratio pre- and postoperatively.

Subsequently, for the analysis of risk factors for hypoglycemia, 19 variables were selected based on previous reports [5,6] and clinical experience: age, body weight, sex, MMVD severity, BCS, MCS, allogeneic blood transfusion, blood collection for autologous blood transfusion, autologous washed RBC transfusion, postoperative opioids, preoperative blood glucose levels, preoperative serum insulin concentrations, preoperative plasma glucagon concentrations, preoperative I/G ratio, hemodilution ratio, in/out balance during CPB, CPB time, cardiac arrest time, and time under anesthesia.

Univariate logistic analysis was performed for each variable, and variables with a *p*-value < 0.2 were selected. The variance inflation factor (VIF) was calculated for each selected variable, considering a VIF ≥ 4 as indicative of multicollinearity. In the presence of multicollinearity, the relationship between risk factors was evaluated using Spearman’s rank correlation index, retaining only one variable (the one with the highest Wald chi-square value from the univariate analysis) for variable pairs with an absolute correlation coefficient |r| > 0.7. The remaining variables were entered into a backward stepwise multivariate logistic analysis, with statistical significance set at a *p*-value < 0.05.

All statistical analyses were performed using IBM SPSS Statistics version 28 (IBM Corp., Armonk, NY, USA).

## 3. Results

### 3.1. Dogs

**Figure 1:**
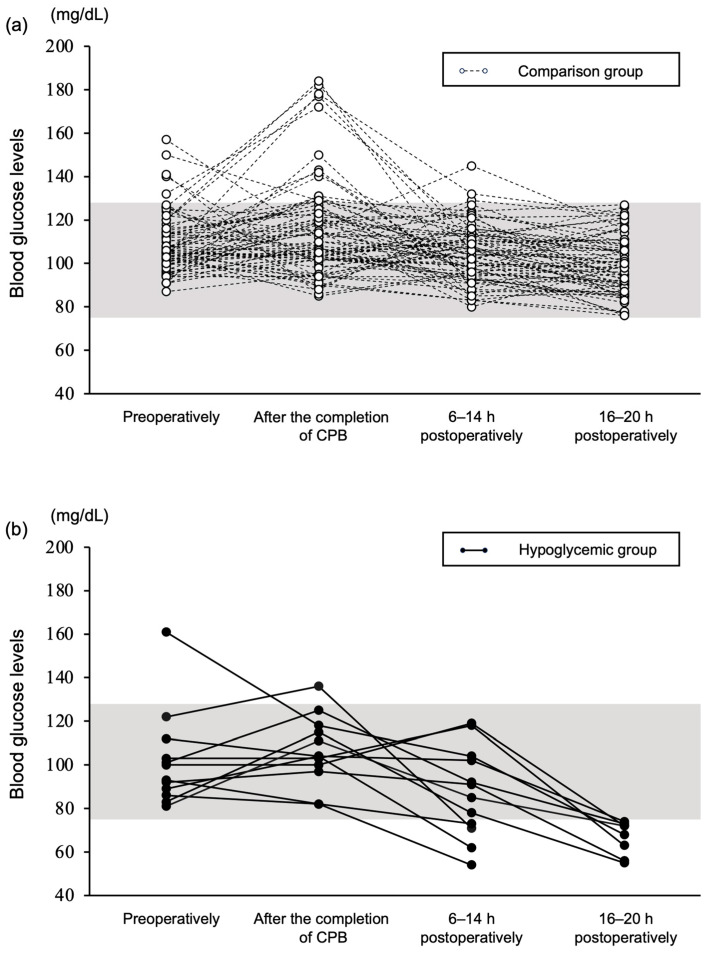
Blood glucose levels at the four timepoints. Changes in blood glucose levels at the four timepoints in the comparison group (**a**) and hypoglycemic group (**b**) are shown. The hypoglycemic group is indicated by ● and solid lines, and the comparison group by ○ and dotted lines. The comparison group refers to the dogs that did not develop postoperative hypoglycemia. The gray area represents the reference range of blood glucose levels.

Eighty-four dogs were included in this study. All dogs remained stable until the last data collection point, and none exhibited signs of suspected respiratory or circulatory failure. The changes in blood glucose levels at the four timepoints are shown in Figure 1. The dogs were divided into the hypoglycemic or the comparison (dogs that did not develop hypoglycemia) group. Some dogs showed elevated glucose levels preoperatively, but the levels were in the mild range [15]. The age, body weight, sex, MMVD severity, BCS, and MCS of the dogs are presented in Table 1.

The investigated dog breeds, in order of the highest occurrence, were Chihuahua (*n* = 31), Toy Poodle (*n* = 8), Mix (*n* = 8), Maltese (*n* = 8), Cavalier King Charles Spaniel (*n* = 4), Yorkshire Terrier (*n* = 4), Shih Tzu (*n* = 4), Pomeranian (*n* = 4), Miniature Schnauzer (*n* = 3), and Papillon (*n* = 2). Other breeds included Norfolk Terrier, Bichon Frise, Miniature Pinscher, Jack Russell Terrier, Dachshund, Lhasa Apso, Chinese Crested, and American Cocker Spaniel (*n* = 1 each). The breeds that developed postoperative hypoglycemia were Chihuahua (*n* = 8), Maltese (*n* = 2), Yorkshire Terrier (*n* = 1), and Shih Tzu (*n* = 1).

On the day of MVR, all dogs received pimobendan, and 71 dogs received loop diuretics (furosemide, 40 dogs; torasemide, 31 dogs) as cardiovascular medications.

The data related to MVR and CPB are presented in Table 2.

### 3.2. Blood Glucose Levels

There were no cases of hypoglycemia preoperatively or after the completion of CPB. A total of 12 of the 84 dogs (14.2%) experienced hypoglycemia. Hypoglycemia occurred in 4 of the 84 dogs (4.8%) between 6 and 14 h postoperatively and in 8 of 80 dogs (10.0%) between 16 and 20 h postoperatively (Figure 1). In cases of hypoglycemia, the dogs were fed if they had an appetite. If the dog could only drink water, a 50% glucose solution was orally administered. If oral administration was not possible, a 25% glucose solution was administered intravenously. Following these treatments, blood glucose levels improved.

### 3.3. Insulin, Glucagon, and Insulin/Glucagon Ratio

**Figure 2:**
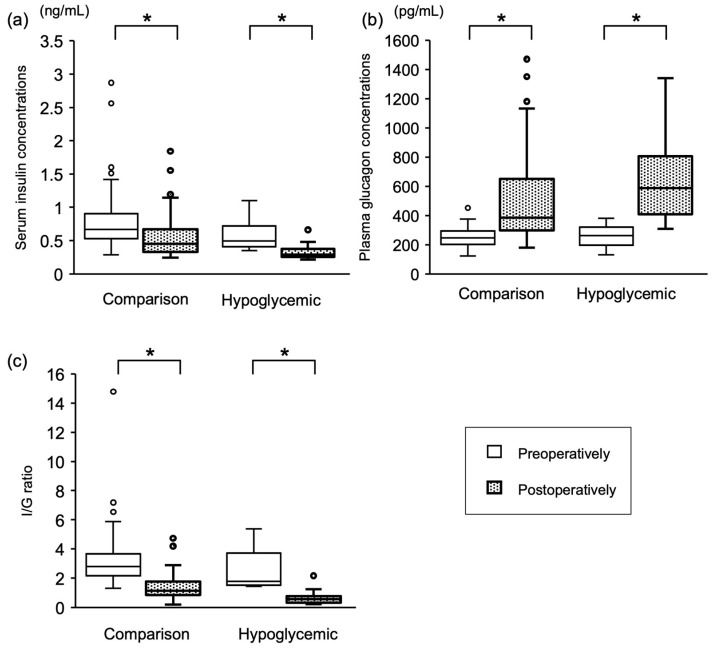
Serum insulin concentrations, plasma glucagon concentrations, and I/G ratios in dogs. The comparison group refers to the dogs that did not develop postoperative hypoglycemia. ○ represent outliers, which are defined as values exceeding 1.5 times of the IQR range. This figure illustrates the changes in insulin concentrations (**a**), glucagon concentrations (**b**), as well as I/G ratios (**c**) between the preoperative and postoperative periods. In both groups, the postoperative serum insulin concentrations and I/G ratios decreased, whereas the plasma glucagon concentrations increased. I/G: insulin/glucagon *: *p* < 0.05.

Both the hypoglycemic and comparison groups had significantly lower insulin concentrations (*p* = 0.002 and *p* < 0.001, respectively) and significantly higher glucagon concentrations (*p* < 0.001, both) postoperatively. The I/G ratio decreased significantly postoperatively in both groups (*p* = 0.002 and *p* < 0.001, respectively; Figure 2).

### 3.4. Risk Factors of Hypoglycemia

In the univariate logistic analysis, 12 variables were selected as risk factor candidates (Table 3).

Variables with multicollinearity were excluded using correlation coefficients. After exclusion, 10 variables remained, and no multicollinearity was observed among these variables (Table 3). The final multivariate analysis model included 10 variables, with only body weight and MMVD severity as significant risk factors (Table 4).

## 4. Discussion

To the best of the authors’ knowledge, this is the first study to investigate the potential occurrence of postoperative hypoglycemia after MVR in dogs. Although hypoglycemia was not present in all dogs preoperatively, it occurred in 14.2% of cases after MVR. In this study, the authors focused on insulin and glucagon, which play significant roles in blood glucose regulation, and examined changes in these two hormones pre- and postoperatively. The hypoglycemic group showed lower insulin and higher glucagon levels postoperatively than preoperatively, leading to a reduced I/G ratio. Therefore, the cause of postoperative hypoglycemia did not appear to be hyperinsulinemia or hypoglucagonemia.

Hypoglycemia occurred most frequently at 16–20 h postoperatively. Combining the preoperative fasting period and this postoperative period resulted in an approximately 30 h fasting period. Typically, glycogenolysis and gluconeogenesis increase glucose entry into circulation and prevent a decrease in blood glucose levels [16]. Nevertheless, in cases where hypoglycemia occurred, this regulatory mechanism may not have functioned appropriately. In humans, some reports of postoperative hypoglycemia have suggested that liver glycogen depletion and dysfunction of gluconeogenic pathways may occur because of malnutrition [6]. Furthermore, the authors conducted an analysis that included BCS and MCS, which are indicators of malnutrition (cachexia) in dogs [17]. However, as these two factors were not identified as significant risk factors, an association between malnutrition and postoperative hypoglycemia is unlikely.

This study indicates that low body weight is a risk factor for postoperative hypoglycemia. Most of the dogs that developed postoperative hypoglycemia were Chihuahuas. The large proportion of Chihuahuas in this study population and the relatively low body weight in this breed could be possible explanations. However, no clear conclusions can be drawn owing to the small sample size. Animals with lower body weight typically have a higher surface area-to-volume ratio, leading to increased heat loss and a greater risk of postoperative hypothermia [18,19]. Moreover, Kleiber’s law states that energy consumption is proportional to the three-fourth power of the body weight [20], meaning that animals with a lower body weight may have a higher glucose consumption rate per unit of body weight. In a hypothermia setting, energy consumption may increase postoperatively for heat production by shivering and adipose tissue burning calories [21]. The authors’ anesthetic method for MVR combines hypothermia and inhalation of gas. Usually, the authors rewarm the dog up to 37 °C before completion of CPB. However, the authors have commonly experienced a decrease in body temperature at the end of surgery. Although no data were available for postoperative body temperature in this study, there may have been a connection between postoperative body temperature and hypoglycemia.

Another risk factor of postoperative hypoglycemia is asymptomatic MMVD. The authors predicted that dogs with severe MMVD, which often experience general deterioration, would be more prone to hypoglycemia. The finding of this study that asymptomatic dogs were more likely to experience hypoglycemia was unexpected. There may be factors that make symptomatic dogs less susceptible to glucose reduction. Additionally, elevated endogenous catecholamine levels resulting from an activated sympathetic nervous system have been reported in dogs with heart failure [22,23], and enhanced cortisol secretion has been reported in humans with heart failure [24]. These can induce insulin resistance and increase blood glucose levels. In humans, insulin resistance may occur because of heart failure [25]. The present study did not investigate such hormones or insulin resistance, warranting future research on the matter. Additionally, loop diuretics may affect blood glucose levels. High-dose loop diuretics are generally required in dogs with symptomatic MMVD. Furthermore, loop diuretics can lower both intra- and extracellular potassium levels, which may lead to the inhibition of insulin secretion and decreased peripheral insulin sensitivity, potentially resulting in elevated blood glucose levels [26]. These changes induced by heart failure may explain why symptomatic dogs are less likely to experience postoperative hypoglycemia.

Other factors potentially involved in blood glucose regulation include sex hormones, hypoxia, and perioperative medications. Sex was included as a variable to investigate the role of sex hormones; however, it was not a significant risk factor in this analysis. Under hypoxic conditions, animals may experience increased glucose consumption, potentially leading to hypoglycemia [27]. Two factors that could lead to postoperative hypoxia are present in dogs undergoing MVR: (1) dogs with left heart failure may have reduced lung oxygenation capacity, and the added stress of thoracic surgery can further compromise it, and (2) blood dilution and red blood cell destruction caused by CPB and bleeding potentially cause postoperative anemia, resulting in tissue hypoxia. However, none of the dogs in this study showed signs of respiratory failure during postoperative management, indicating that factor (1) is not likely. Moreover, variables related to blood dilution and anemia, including the hemodilution ratio, in/out balance during CPB, presence of allogeneic blood transfusions, and presence of autologous washed RBC transfusions, were considered as factors; however, none proved to be a significant risk factor, meaning that factor (2) is also less likely. Therefore, it is unlikely that hypoxia contributes significantly to hypoglycemia. The authors considered the possibility of medications used during the perioperative period being related to hypoglycemia. All dogs in this study were on pimobendan, which has insulinotropic properties [28], but the postoperative decrease in insulin levels implies lack of excessive insulinotropic actions. ACD-A was used as an anticoagulant during blood collection for autologous blood transfusion, and MAP was used as a blood preservation solution for allogeneic blood. Both solutions contained glucose. Thus, the authors considered the possibility that autologous blood transfusion and allogeneic blood transfusion could affect postoperative blood glucose control and included these as variables. However, the presence or absence of autologous blood transfusion and allogeneic blood transfusion was not a significant risk factor, suggesting that the influence of the ACD-A and MAP solutions on blood glucose control is likely minimal. Intraoperative hypotension was treated with crystalloid fluid and a low dose of dobutamine. Low-dose dobutamine is considered to have a minimal impact on blood glucose levels [29,30]. Catecholamines (such as adrenaline and noradrenaline), which strongly affect glucose regulation, were not administered. Long-term opioid use has also been associated with decreased cortisol responsiveness to hypoglycemia in humans [31]. Postoperative opioid use was included as a variable but was not a significant risk factor for hypoglycemia. Therefore, it is likely that the drugs administered during the perioperative period had minimal effects on blood glucose control.

This study had some limitations. First, the number of dogs with hypoglycemia (*n* = 12) was small for the variables in the multivariate analysis. This could have lessened the stability of the results of the multivariate analysis. Second, hypoglycemia was defined as a glucose level of <75 mg/dL. However, the threshold for hypoglycemia varies from 60 to 75 mg/dL across studies [14,27,32,33]. The authors chose a 75 mg/dL threshold based on the reference value set by the measuring machine, which may have resulted in an overestimation of hypoglycemia. Nevertheless, it is meaningful to note that some dogs with blood glucose levels above this threshold preoperatively experienced a drop below this threshold postoperatively. Third, the blood glucose levels were measured at specific timepoints and not continuously. Therefore, the detection of hypoglycemia was possibly delayed. Fourth, this study lacks control groups, including a group of healthy dogs corresponding to the fasting periods of the cases in this study and a control group of dogs undergoing other types of open-chest surgery, that could be used for comparison of the incidence of postoperative hypoglycemia. The lack of these groups precluded determining that the hypoglycemia was truly attributable to MVR.

## 5. Conclusions

This study showed that hypoglycemia can occur after open-heart surgery in dogs. In dogs that developed hypoglycemia after MVR, plasma glucagon levels were higher and serum insulin levels were lower than the preoperative levels, indicating that the cause of postoperative hypoglycemia did not appear to be hyperinsulinemia or hypoglucagonemia. Low body weight and asymptomatic MMVD were indicated as risk factors for hypoglycemia after MVR. Monitoring blood glucose levels after MVR should be included in the standard hospitalization plan to prevent hypoglycemic emergencies in dogs.

## Figures and Tables

**Table 1:** Epidemiologic characteristics of the dogs in the hypoglycemic and comparison groups.

Characteristics	Hypoglycemic (*n =* 12)	Comparison (*n* = 72)
Age (years)	10 (2)	10 (2)
Body weight (kg)	2.4 (2.2–2.7)	4.1 (3.1–5.8)
Sex (F/FS/M/MC)	(0/6/1/5)	(3/27/11/31)
MMVD severity (asymptomatic/symptomatic)	(7/5)	(20/52)
BCS (1–9)	5 (5–6)	5 (4–6)
MCS (1–4)	4 (4–4)	4 (3–4)

The obtained data are from the hypoglycemic and comparison groups and presented as mean (SD) or median (IQR). The comparison group refers to the dogs that did not develop postoperative hypoglycemia. BCS: body condition score, F: female, FS: female spayed, M: male, MC: male castrated, MCS: muscle condition score, MMVD: myxomatous mitral valve disease.

**Table 2:** Characteristics of factors related to MVR.

Characteristics	Hypoglycemic (*n* = 12)	Comparison (*n* = 72)
Allogeneic blood transfusion (yes/no)	9/3	20/52
Blood collection for autologous blood transfusion (yes/no)	6/6	61/11
Autologous washed RBC transfusion (yes/no)	3/9	16/56
Postoperative opioid utilization (yes/no)	11/1	58/14
Hemodilution rate (%)	36.7 (35.7–40.7)	30.5 (7.3)
In–out fluid balance during CPB (mL/kg)	49.6 (25.0)	27.7 (19.1–42.3)
CPB time (min)	78 (74–81)	82 (75–92)
Cardiac arrest time (min)	47 (43–53)	51 (46–58)
Time under anesthesia (min)	200 (31)	210 (195–235)

The obtained data are from the hypoglycemic and comparison groups and presented as mean (SD) or median (IQR). The comparison group refers to the dogs that did not develop postoperative hypoglycemia. CPB: cardiopulmonary bypass, RBC: red blood cell.

**Table 3:** Univariate logistic regression analysis in dogs.

Characteristics	*p*-Value	VIF	Adjusted VIF
Age	0.488	-	-
Body weight	0.009 *	6.835	2.229
Sex	0.333	-	-
MMVD severity	0.044 *	1.110	1.098
BCS	0.570	-	-
MCS	0.109 *	1.263	1.085
Allogeneic blood transfusion	0.004 *	1.581	1.546
Blood collection for autologous blood transfusion	0.010 *	1.624	1.621
Autologous washed RBC transfusion	0.831	-	-
Postoperative opioids	0.369	-	-
Preoperative blood glucose levels	0.135 *	1.231	1.064
Preoperative serum insulin concentrations	0.099 *	5.378	1.283
Preoperative plasma glucagon concentrations	0.592	-	-
Preoperative I/G ratio	0.158 *	4.776	-
Hemodilution rate	0.012 *	4.566	-
In–out fluid balance during CPB	0.058 *	1.790	1.686
CPB time	0.208	-	-
Cardiac arrest time	0.084 *	2.100	1.899
Time under anesthesia	0.122 *	1.575	1.565

*: *p* < 0.2. For variables with a VIF ≥ 4, one variable from the strongly correlated pairs was removed. The VIF after the variable adjustment is referred to as the adjusted VIF. BCS: body condition score, CPB: cardiopulmonary bypass, I/G: insulin/glucagon, MCS: muscle condition score, MMVD: myxomatous mitral valve disease, RBC: red blood cell, VIF: variance inflation factor.

**Table 4:** The final model results of the multivariate logistic regression analysis.

Characteristics	Adjusted OR	95% CI	*p*-Value
Body weight	0.288	0.115–0.720	0.008 *
MMVD severity, symptomatic (vs. asymptomatic)	0.183	0.043–0.788	0.023 *

*: *p* < 0.05. Adjusted odds ratios for the occurrence of hypoglycemia in symptomatic versus asymptomatic dogs for MMVD severity. CI: confidence interval, MMVD: myxomatous mitral valve disease, OR: odds ratio.

## Data Availability

The raw data supporting the conclusions of this study will be made available by the authors on request.
